# O-DEM: ein neues kognitives Screening bei Schwerhörigkeit

**DOI:** 10.1007/s00106-023-01293-y

**Published:** 2023-04-24

**Authors:** I. Ballasch, A. De Kruif, M. K. Hendel, C. Rohr, I. Brünecke, E. Kalbe, C. Völter, J. Kessler

**Affiliations:** 1 Klinik und Poliklinik für Neurologie, Uniklinik Köln, Köln, Deutschland; 2 Klinik für Hals‑, Nasen- und Ohrenheilkunde, Kopf- und Halschirurgie, Katholisches Klinikum, Ruhr-Universität Bochum, Bochum, Deutschland; 3 Medizinische Psychologie I Neuropsychologie und Gender Studies, Centrum für neuropsychologische Diagnostik und Intervention (CeNDI), Medizinische Fakultät und Uniklinik Köln, Universität zu Köln, Köln, Deutschland

**Keywords:** Kognitiver Test, Kognitive Beeinträchtigung, Demenz, Hörverlust, Demenzscreening, Cognitive test, Cognitive impairment, Dementia, Hearing impairment, Dementia screening

## Abstract

**Hintergrund:**

Schwerhörigkeit ist ein relevanter Risikofaktor einer Demenz. Bisher kann eine kognitive Beeinträchtigung oder Demenz von Personen mit Hörbeeinträchtigungen (HB) wegen des eingeschränkten Sensoriums von gängigen kognitiven Screeningverfahren nicht adäquat erfasst werden. Deshalb besteht ein Bedarf eines solchen an Hörgeschädigte angepassten Screenings. Ziel der Studie war es, ein kognitives Screening für Menschen mit HB zu entwickeln und zu evaluieren.

**Material und Methoden:**

Hierzu wurden drei Aufgaben, eine Wortflüssigkeitsaufgabe, der Trail Making Test A (TMT-A) und eine Subtraktionsaufgabe in einem neuen kognitiven Screening, dem O‑DEM, zusammengefasst. Dieser wurde in einem ersten Schritt an 2837 Patient*innen ohne subjektive HB und anschließend an 213 Patient*innen mit objektiv erfassten HB evaluiert und mit dem Hearing-Impaired Montreal Cognitive Assessment (HI-MoCA) verglichen.

**Ergebnisse:**

Es zeigte sich, dass jeder O‑DEM-Subtest signifikant zwischen keinen, leichten und mittleren bis ausgeprägten kognitiven Beeinträchtigungen unterscheiden kann. Basierend auf dem Mittelwert und der Standardabweichung der Menschen ohne kognitive Beeinträchtigungen wurde eine Transformation der Rohwerte vorgenommen und ein Gesamtscore mit einer maximalen Ausprägung von 10 festgelegt. Ebenso zeigte die Untersuchung an den hörgeschädigten Patient*innen, dass der O‑DEM genauso sensitiv wie der HI-MoCA zwischen Menschen mit und ohne kognitive Beeinträchtigungen differenzieren kann.

**Schlussfolgerungen:**

Der O‑DEM ist gegenüber anderen Verfahren ein vergleichbar schnell durchzuführendes Screening zur Detektion von leichten und mittleren kognitiven Beeinträchtigungen bei Menschen mit HB.

## Abstract (en)

**Background:**

Hearing loss is a significant risk factor for dementia. To date, cognitive impairment and dementia in patients with hearing impairment (HI) cannot be adequately diagnosed by commonly administered cognitive screening tests due to sensory impairments. Therefore, an adapted screening is needed. The aim of the present study was to develop and evaluate a cognitive screening for people with HI.

**Materials and methods:**

The new cognitive screening, called O‑DEM, entails a word fluency test, the Trail Making Test A (TMT-A), and a subtraction task. First, the O‑DEM was tested in a large clinical sample (*N* = 2837) of people without subjective HI. In a second step, the O‑DEM was evaluated in 213 patients with objectively assessed HI and compared with the Hearing-Impaired Montreal Cognitive Assessment (HI-MoCA).

**Results:**

The results indicate that the O‑DEM subtests significantly discriminate between participants with no, mild, and moderate to severe cognitive impairment. Based on the mean and standard deviation of the participants without cognitive impairment, a transformation of the raw scores was performed and a total score with a maximum value of 10 was determined. In the second part of the study, the O‑DEM was shown to be as sensitive as the HI-MoCA in differentiating between people with and without cognitive impairment.

**Conclusion:**

Compared to other screenings, the O‑DEM is a quickly administrable screening for the detection of mild and moderate cognitive impairment in people with HI.

Kognitive Screenings im Rahmen der Demenzdiagnostik sind häufig auditiv basiert und somit für Menschen mit Hörbeeinträchtigungen (HB) ungeeignet. Die Zunahme älterer Menschen mit auditiven und kognitiven Beeinträchtigungen verdeutlicht die Notwendigkeit eines Screenings, das HB berücksichtigt und im klinischen Alltag praktikabel ist.

Die heutigen gesellschaftlichen Veränderungen und medizinischen Fortschritte gehen mit einer höheren Lebenserwartung einher, welche zu einem Anstieg alterskorrelierter Krankheiten führt. So beläuft sich die Prävalenz demenzieller Erkrankungen in Deutschland derzeit auf ca. 1,5 Mio. Menschen [5].

Neben verminderten kognitiven Leistungen treten Einschränkungen des Hörvermögens im Alter bei etwa 20 % der 60- bis 69-Jährigen und 40 % der 70- bis 79-Jährigen in Deutschland auf [22]. Dabei scheint ein altersassoziierter Hörverlust ein bedeutsamer Risikofaktor einer Demenz zu sein, der bei frühzeitiger Behandlung möglicherweise eine positive Auswirkung auf die Kognition im Alter hat [13].

Berücksichtigt man das erhöhte Risiko Hörgeschädigter für die Entwicklung einer Demenz und die Häufigkeit einer Schwerhörigkeit im Alter, ist es überraschend, dass bisher erst vereinzelt eine Adaption kognitiver Screeningverfahren erfolgte.

Das kognitive Potenzial von Menschen mit Höreinschränkungen wird in Standardtests wie dem Montreal Cognitive Assessment (MoCA; [14]), Demenz-Detektions-Test (DemTect; [7]) und Mini Mental Status Test (MMST; [9]), die intaktes Sehen und Hören voraussetzen, oftmals unterschätzt (z. B. [6]). Bei schwerhörigen Patient*innen kann sich die Testleitung nicht sicher sein, ob eine fehlerhafte Bearbeitung der Aufgabe auf mangelnde kognitive Fähigkeiten, missverstandene Instruktionen, falsche Item-Wahrnehmungen oder erhöhte Ablenkung durch eine geänderte Hörfähigkeit zurückzuführen ist. Bei bis zu 16 % aller Proband*innen mit HB wird fälschlicherweise eine Demenz diagnostiziert [21].

Zuletzt wurden vor allem ausführliche Testbatterien wie die Repeatable Battery for the Assessment of Neuropsychological Status für hörgeschädigte Personen [3], die Cambridge Neuropsychological Test Automated Battery [17] und die computerbasierte neurokognitive Testbatterie ALAcog [23] für Schwerhörige entwickelt. Durch ihren Umfang sind sie allerdings weitestgehend Forschungszwecken vorbehalten.

Ein altersassoziierter Hörverlust scheint ein bedeutsamer Risikofaktor einer Demenz zu sein

Vereinzelte Adaptionen bestehender neuropsychologischer Screenings für Personen mit auditiven Einschränkungen sind der HI-MoCA [12], der MoCA Hearing Impaired version (MoCA-HI; [4]) der MMST für Hörgeminderte [19] sowie der DemTect Eye+Ear [8]. Aufgrund mangelnder Sensitivität und fehlender Validierungsstudien können lediglich der HI-MoCA, MoCA-HI und DemTect Eye+Ear als adäquate kognitive Screeningverfahren für Menschen mit Hör- und Sehminderung genutzt werden. Bislang fehlen allerdings Normdaten für den deutschsprachigen Raum, zudem sind sie hauptsächlich neurologischen Praxen vorbehalten. Die Durchführungszeit (15, DemTect Eye+Ear, bis zu 20 min, HI-MoCA) ist zeit- und kostenintensiv. Die Komplexität der Aufgaben erfordert ausreichende Kenntnisse zur Testdurchführung, an denen es in herkömmlichen Praxen oftmals mangelt und Verlaufsuntersuchungen erschwert.

Demzufolge besteht ein großer Bedarf an modifizierten Screeningverfahren zur Erkennung kognitiver Beeinträchtigungen bei Schwerhörigen, welcher mithilfe des O‑DEM (ein Akronym für Ohren und Demenz) gedeckt werden soll. Da deutsche Testkennwerte (Validität, repräsentative Stichprobe etc.) für Menschen mit HB fehlen, entstand der O‑DEM auf Basis der zur Verfügung stehenden Tests zur Untersuchung relevanter kognitiver Domänen. Die vorliegende Studie besteht aus zwei Teilen. Zu Beginn wurde der O‑DEM an Menschen ohne selbstberichtete HB (erstes Kollektiv) getestet, um erste Richtwerte zu entwickeln. Im Fokus stand die Frage, wie sensitiv der O‑DEM zwischen Menschen mit keinen, leichten und mittleren bis ausgeprägten kognitiven Beeinträchtigungen unterscheiden kann. Anschließend wurde der O‑DEM an einer Stichprobe von Menschen mit objektiven HB (zweites Kollektiv) validiert und mit dem HI-MoCA verglichen.

## Methode

### O-DEM – Screening

**Figure 1:**
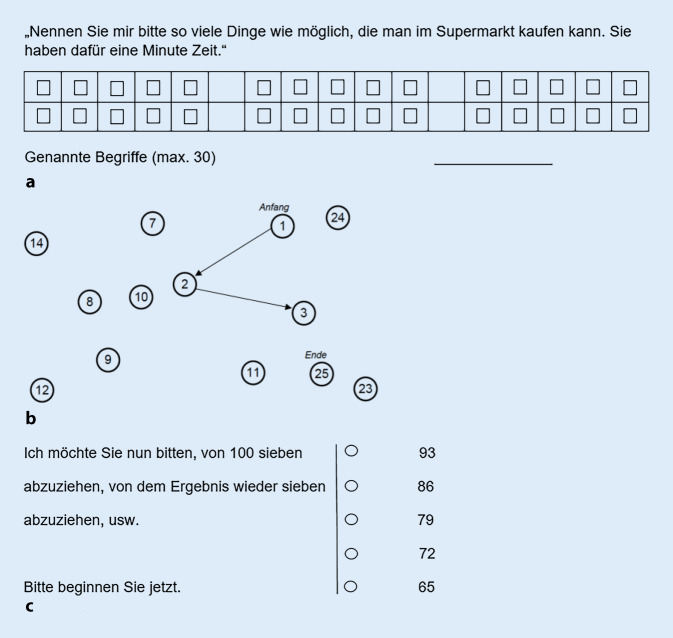

Der O‑DEM ist ein einfach und schnell durchführbares Demenzscreening für Menschen mit HB und besteht aus drei Tests bereits validierter und international bewährter neuropsychologischer Batterien: Supermarktaufgabe [7], TMT‑A [16] und Subtraktionsaufgabe ([9]; Abb. 1).

Der O‑DEM ist ein einfach und schnell durchführbares Demenzscreening

Die drei Subtests wurden anhand von acht Kriterien ausgewählt: hohe Sensitivität [11]; objektive Durchführbarkeit und Auswertung; Durchführbarkeit bei älteren Menschen mit kognitiven Beeinträchtigungen; Verlaufsuntersuchungen möglich und dokumentierbar über Zeit; Ökonomie (zeit- und kostensparend); Durchführbarkeit und Auswertung durch einfache Delegierbarkeit und Verständlichkeit ohne psychologisch geschultes Fachpersonal; einfache visuell vermittelbare Instruktionen; Messung relevanter kognitiver Domänen. Die Domänenauswahl resultierte aus einem Vergleich verfügbarer Subtests, der die ausgewählten O‑DEM-Subtests als am diskriminativsten identifizierte.

Die Instruktionen wurden auf Tafeln in gut lesbarer Schrift (Arial zwölf) präsentiert. Die originalen auditiven Instruktionen wurden unverändert ins Visuelle übertragen. Die Beschäftigungszeit mit den Instruktionen war pro Patient*in variabel, aber insgesamt kurz. Verständnisfragen waren erlaubt. Nur auf ausdrückliche Nachfragen der Patient*innen wurde auditiv zur Gewährleistung des Verständnisses instruiert.

Die **Supermarktaufgabe** aus dem DemTect [7] erfasst Störungen der Wortproduktion, Wortflüssigkeit und des semantischen Gedächtnisses. Innerhalb einer Minute sollen so viele Dinge wie möglich genannt werden, die man im Supermarkt kaufen kann. Jedes richtig genannte Objekt entspricht einem Punkt. Eine höhere Punktzahl stellt eine bessere Leistung dar (Abb. 1a).

Der **TMT‑A** [16] erfasst die kognitive Verarbeitungsgeschwindigkeit und visuoperzeptive Leistung. Auf einer DIN-A4-Seite müssen verstreute Zahlen in aufsteigender Reihenfolge (1–25) möglichst schnell miteinander verbunden werden. Dabei wird die Zeit in Sekunden gemessen; eine längere Bearbeitungszeit bedeutet ein schlechteres Ergebnis (Abb. 1b).

Die **Subtraktionsaufgabe** (‚Serial Seven‘) des MMST [9] dient der Erfassung der Aufmerksamkeit, basalen Rechenfähigkeit und des Arbeitsgedächtnisses. Ausgehend von 100 werden sieben subtrahiert; von dem Folgeergebnis werden erneut sieben subtrahiert. Dieser Vorgang wird fünfmal wiederholt. Für jedes richtige Ergebnis wird ein Punkt vergeben. Je mehr Punkte erreicht werden, desto besser ist die Leistung (Abb. 1c).

### Erster Teil der Studie

#### Stichprobenbeschreibung des ersten Kollektivs

Eingeschlossen wurden die Daten von 4977 Patient*innen, die sukzessive im Zeitraum von 2010 bis 2019 in der AG Neuropsychologie in der Klinik und Poliklinik für Neurologie des Universitätsklinikums Köln im Rahmen der neuropsychologischen Standarddiagnostik untersucht wurden. Gründe des stationären Aufenthalts waren subjektive Beschwerden über kognitive Defizite, eine Überweisung des Hausarztes oder eine ambulante Untersuchung. Bei allen Personen lag die Einwilligungsfähigkeit vor. Die Diagnosen umfassten das ganze Spektrum neurologischer Erkrankungen.

Einschränkungen des Hör- oder Sehvermögens wurden durch anamnestische Angaben der Patient*innen anhand einer sechsstufigen Skala (1 = sehr gut bis 6 = ungenügend) und der klinischen Einschätzung der Testleitung ausgeschlossen. Mangelndes Hör- und Sehvermögen (5 oder 6 Punkte) führte zum Ausschluss. Weitere Ausschlusskriterien umfassten mangelnde Deutschkenntnisse, das Vorliegen einer Betreuung und einer Depression (> 19 von 64 Punkten im Beck-Depressions-Inventar II [1]) sowie weiterer psychiatrischer Erkrankungen.

Gemäß der Einschätzung der Testleitung war es allen Patient*innen möglich, in Zimmerlautstärke zu kommunizieren.

**Figure 2:**
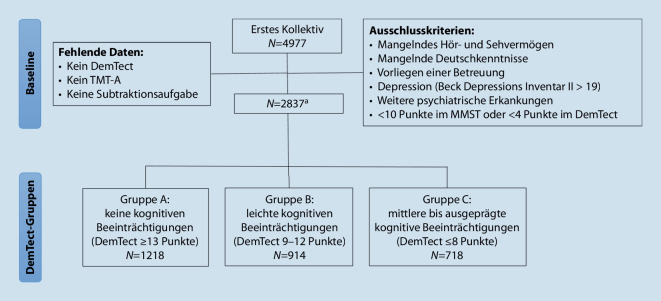

Personen, die mindestens 10 der 30 Punkte im MMST und 4 der 18 Punkte im DemTect erreichten, wurden eingeschlossen. Alter und Geschlecht waren für das Ziel der Studie irrelevant, da der Test für verschiedene Altersgruppen untersucht werden sollte. Nach paarweisem Ausschluss inkompletter Datensätze enthielt die große Stichprobe Patient*innen mit unterschiedlichen Schweregraden kognitiver und mnestischer Einbußen (*N* = 2837; Abb. 2). Untersucht wurden 1583 (55,8 %) Männer (x̄ = 63,96 Jahre, *SD* = 16,0) und 1254 (44,2 %) Frauen (x̄ = 64,09 Jahre, *SD* = 16,1). Von diesen hatten 2,8 % der Patient*innen keinen Schulabschluss, 35,9 % einen Volksschul‑/Hauptschulabschluss, 23,4 % einen Realschulabschluss und 37,9 % das Abitur.

#### Durchführung der Patient*innenuntersuchung

Die Patient*innen erhielten eine ausführliche Anamnese zum mnestischen, kognitiven und affektiven Status mit dem DemTect, MMST, einem Test zur phonematischen Wortflüssigkeit (FAS; [2]), TMT‑A und Trail Making Test B (TMT‑B; [16]), Rey-Osterrieth-Figur-Copy (ROF; [15]) sowie ROF-Recall. Der DemTect beinhaltete die Supermarktaufgabe und der MMST die Subtraktionsaufgabe des O‑DEM. Der TMT‑A ist der dritte Subtest des O‑DEM.

Entsprechend den DemTect-Werten wurden drei Gruppen gebildet: keine kognitiven Beeinträchtigungen (≥ 13 Punkte; *N* = 1218, Referenzgruppe A), leichte kognitive Beeinträchtigungen (9–12 Punkte; *N* = 914, Gruppe B) und mittlere bis ausgeprägte kognitive Beeinträchtigungen (≤ 8 Punkte; *N* = 718, Gruppe C; Abb. 2). Die Bildungsjahre waren in allen drei Gruppen annähernd gleich verteilt.

### Zweiter Teil der Studie

#### Stichprobenbeschreibung des zweiten Kollektivs

**Figure 3:**
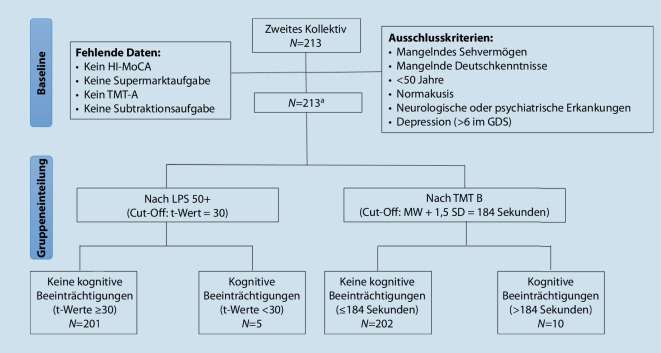

213 Patient*innen wurden im Zeitraum von 2020 bis 2021 in der Klinik für Hals‑, Nasen- und Ohrenheilkunde, Kopf- und Halschirurgie und im Cochlea-Implantat-Zentrum des Katholischen Klinikums der Ruhr-Universität Bochum untersucht. Einschlusskriterien waren ausreichendes Sehvermögen und ausreichende Deutschkenntnisse, ein Alter ab 50 Jahren und eine Hörminderung. Ausschlusskriterien waren eine Normakusis sowie das Vorliegen einer neurologischen oder psychiatrischen Erkrankung und einer Depression (> 6 von 15 Punkten in der Geriatrischen Depressions-Skala (GDS; [18]; Abb. 3)).

Die Stichprobe bestand aus 132 (62 %) Männern (x̄ = 67,45 Jahre, *SD* = 8,8) und 81 (38 %) Frauen (x̄ = 68,95 Jahre, *SD* = 9,3), das mittlere Alter betrug 68,02 Jahre (*SD* = 9,0). Davon hatten 62,4 % (*N* = 133) der Patient*innen mehr als 12 Jahre Bildung im Vergleich zu 37,6 % (*N* = 80) mit 12 oder weniger Jahren.

#### Durchführung der Patient*innenuntersuchung

Neben einem seitengetrennten Reintonaudiogramm in den Frequenzen von 500 bis 4000 Hertz (Hz) wurde der FAS, HI-MoCA [10], die Aufgabe 4 des Leistungsprüfsystems für 50- bis 90-Jährige (LPS 50+; [20]), der TMT‑A und TMT‑B durchgeführt. Für die Gruppenaufteilung in mit und ohne kognitive Beeinträchtigungen wurden diverse Cut-off-Möglichkeiten untersucht. Aufgrund statistischer Evidenz wurden die zwei Varianten, die in Tab. 1 dargestellt sind, ausgewählt. Die HB-Klassen wurden entsprechend der WHO in leichte (26–40 Dezibel, dB), mittelgradige (41–60 dB), schwergradige HB (61–80 dB) und Taubheit (≥ 81 dB) eingeteilt (Abb. 3).

**Table 1:** 

Cut-off	Keine kognitiven Beeinträchtigungen	Kognitive Beeinträchtigungen
LPS 50 + t-Wert = 30	t‑Werte ≥ 30	t‑Werte < 30
TMT‑B MW + 1,5 SD = 184 s	≤ 184 s	> 184 s

*MW* Mittelwert, *SD* Standardabweichung

### Statistische Analysen

Die statistischen Analysen wurden mit SPSS Statistics 26.0 (IBM, Armonk) durchgeführt und das Signifikanzniveau auf α = 0,05 festgesetzt. Im ersten Teil der Studie wurden die deskriptive Statistik und Korrelationen mit zweiseitigem Spearman-Rangkorrelationskoeffizienten für das erste Kollektiv berechnet. Unterschiede der DemTect-Gruppen in den O‑DEM-Subtests wurden mittels univariaten Varianzanalysen und Bonferroni-bereinigtem α‑Niveau von 0,016 pro Test (0,05/3) berechnet. Zudem wurde eine „Receiver-Operating-Characteristic(ROC)-Analyse“ mit den O‑DEM-Subtests durchgeführt. Die Fläche unter der ROC-Kurve (AUC = Area Under the Curve) diente der Test-Qualitätsklassifizierung. Mithilfe der ROC-Analyse wurden die Subtests gewichtet und basierend auf dem Mittelwert und der Standardabweichung für die Referenzgruppe A eine Transformation der jeweiligen O‑DEM-Rohwerte vorgenommen. Insgesamt wurden zehn Punkte vergeben, die Supermarktaufgabe und der TMT‑A wurden basierend auf der ROC-Analyse mit vier Punkten und die Subtraktionsaufgabe mit zwei Punkten eingeordnet. Auch wurde die AUC für den O‑DEM-Gesamtscore bestimmt. Im zweiten Teil der Studie wurde die deskriptive Statistik für das zweite Kollektiv und die HB-Gruppen sowie Korrelationen des O‑DEMs mit den kognitiven Tests, Alter, Bildung und Hören berechnet. Das Hören ergab sich aus der gemittelten HB in dB bei 500, 1000, 2000 und 4000 Hz für das linke sowie das rechte Ohr. ROC-Analysen wurden für den O‑DEM-Gesamtscore und HI-MoCA-Gesamtscore durchgeführt. Die Klassifikation in kognitiv auffällig vs. unauffällig erfolgte entsprechend der LPS 50+- und TMT-B-Werte (s. Methode).

## Ergebnisse

### Erster Teil der Studie

Die Tab. 2 zeigt die deskriptive Statistik jeder DemTect-Gruppe in der neuropsychologischen Testbatterie.

**Table 2:** 

	Alter	Geschlecht	DemTect	MMST	FAS	TMT‑A	TMT‑B	ROF-Copy	ROF-Recall
A	60,28 (16,5)	568 W 645 M	15,24 (1,9)	28,51 (1,7)	33,65 (11,4)	51,61 (31,9)	125,48 (78,2)	33,42 (11,3)	15,94 (7,5)
B	67,03 (15,2)	410 W 498 M	10,6 (1,1)	26,57 (2,8)	24,05 (10,9)	77,1 (51,3)	194,95 (109,8)	28,87 (13,6)	10,1 (9,9)
C	66,59 (1,1)	276 W 440 M	5,73 (2,0)	23,27 (4,3)	15,72 (9,2)	121,99 (87,8)	243,43 (125,7)	24,56 (10,7)	6,51 (6,5)

*A*: keine kognitiven Beeinträchtigungen, *B*: leichte kognitive Beeinträchtigungen, *C*: mittlere bis ausgeprägte kognitive Beeinträchtigungen, *W* weiblich, *M* männlich, *DemTect*: Maximum (Max.) 18 Punkte, *MMST*: Max. 30 Punkte, *FAS*: ≥ 30 Wörter nicht pathologisch, *TMT‑A*: in Sekunden, Perzentil 50 = 37 s, *TMT‑B*: in Sekunden, *ROF-Copy*: Max. 36 Punkte, *ROF-Recall*: Max. 36 Punkte

Die Korrelationen der O‑DEM-Subtests mit dem DemTect-Gesamtscore, MMST-Gesamtscore, Alter und Bildung waren signifikant (*p* < 0,001; Tab. 3).

**Table 3:** 

Variabel	DemTect	MMST	Alter	Bildung
Supermarktaufgabe	0,71*	0,53*	−0,32*	0,17*
TMT‑A	−0,49*	−0,53*	0,36*	−0,15*
Subtraktionsaufgabe	0,47*	0,72*	−0,10*	0,16*

*p < 0,001; zweiseitig

Eine Varianzanalyse der drei Subtests mit nachfolgender Bonferroni-Korrektur zeigte signifikante Unterschiede zwischen allen Gruppen beim Subtest Supermarkt, *F*(2,1) = 1772,2; *p* < 0,001; beim TMT‑A, *F*(2,1) = 338,44; *p* < 0,001; und bei der Subtraktionsaufgabe, *F*(2,1) = 604,57; *p* < 0,001. In allen drei Subtests erreichte Gruppe A eine bessere Leistung im Vergleich zu Gruppe B; Gruppe B erzielte eine bessere Leistung als Gruppe C.

In der ROC-Analyse wurden zur Bestimmung der Sensitivität und Spezifität die DemTect-Gruppen A und C sowie A und B hinsichtlich der O‑DEM-Subtests verglichen (Tab. 4).

**Table 4:** 

Gruppenvergleich	Supermarkt	TMT‑A	Subtraktion	O‑DEM-Gesamtwert
A vs. C	0,94	0,82	0,77	0,92
A vs. B	0,77	0,70	0,58	0,76

*A*: keine kognitiven Beeinträchtigungen, *B*: leichte kognitive Beeinträchtigungen, *C*: mittlere bis ausgeprägte kognitive Beeinträchtigungen

Nach der Transformation erreichte die Referenzgruppe A einen mittleren Gesamtscore von 8,58 (*SD* = 1,6), Gruppe B von 6,66 (*SD* = 2,2), und Gruppe C von 4,17 (*SD* = 2,5). Legt man einen Cut-off-Wert von 6 zugrunde, sind nur 28 % der Patient*innen der mittleren bis ausgeprägten kognitiv Beeinträchtigten in dieser Gruppe vertreten, während 93 % der Referenzgruppe A diesen Punktwert erreichen. Bei einem Cut-off von 7 sind es 20 % respektive 86 % der Referenzgruppe A (Tab. 5). Die AUC des O‑DEM-Gesamtscores sind Tab. 4 zu entnehmen.

**Table 5:** 

Cut-off	Referenzgruppe A [%]	Mittlere bis ausgeprägte kognitive Beeinträchtigung [%]
6	93	28
7	86	20

*Referenzgruppe A*: keine kognitiven Beeinträchtigungen. Ein Cut-off von 6 Punkten klassifiziert 93 % der Patient*innen ohne kognitive Beeinträchtigung als solche und 28 % als Patient*innen mit mittleren kognitiven Beeinträchtigungen

### Zweiter Teil der Studie

Die Tab. 6 zeigt die deskriptive Statistik des zweiten Kollektivs, Tab. 7 die der HB-Gruppen. In Tab. 8 wird die deskriptive Statistik des O‑DEM-Gesamtscores sowie der O‑DEM-Subtests des ersten Kollektivs (ohne subjektive HB) und zweiten Kollektivs (mit objektiven HB) dargestellt.

**Table 6:** 

Test	Mittelwert (SD)
Hören rechts	50,83 (25,2)
Hören links	50,36 (23,8)
O‑DEM-Gesamtscore	8,11 (1,9)
O‑DEM Supermarkt	3,49 (0,8)
O‑DEM TMT‑A	2,95 (1,2)
O‑DEM Subtraktion	1,53 (0,8)
HI-MoCA	24,38 (2,9)
TMT‑B	113,50 (47,2)
LPS 50+	50,14 (10,0)
FAS	34,25 (10,7)
GDS	1,39 (1,2)

*SD* Standardabweichung, *Hören*: entspricht der durchschnittlichen HB in dB bei 500, 1000, 2000 und 4000 Hz, *HI-MoCA*: *Max*. 30 Punkte, *TMT‑B*: in Sekunden, *LPS 50+*: ≥ 30 nicht pathologisch, *FAS*: ≥ 30 nicht pathologisch, *GDS*: Max. 15 Punkte

**Table 7:** 

	Mittelwert (SD)			
Test	Leichte HB (*N* = 23)	Mittelgradige HB (*N* = 92)	Hochgradige HB (*N* = 69)	Taubheit (*N* = 29)
O‑DEM-Gesamtscore	8,26 (1,7)	8,09 (2,0)	8,20 (1,9)	7,86 (2,3)
O‑DEM Supermarkt	3,35 (1,0)	3,49 (0,8)	3,61 (0,7)	3,29 (0,9)
O‑DEM TMT‑A	3,30 (0,9)	2,90 (1,2)	2,88 (1,2)	2,96 (1,2)
O‑DEM Subtraktion	1,61 (0,7)	1,49 (0,8)	1,57 (0,8)	1,52 (0,7)
HI-MoCA	24,91 (3,2)	24,51 (3,2)	24,25 (2,6)	23,83 (2,6)
TMT‑B	102,83 (22,3)	114,46 (49,2)	117,55 (55,3)	109,14 (31,7)
LPS 50+	49,14 (10,2)	51,55 (10,4)	50,30 (8,0)	46,17 (12,0)
FAS	32,91 (9,6)	36,51 (11,7)	32,94 (9,5)	31,28 (10,0)
GDS	1,35 (1,2)	1,30 (1,2)	1,36 (1,1)	1,76 (1,5)

*SD* Standardabweichung, *leichte HB*: 26–40 dB, *mittelgradige HB*: 41–60 dB, *schwergradige HB*: 61–80 dB, *Taubheit*: ≥ 81 dB, *HI-MOCA*: *Max*. 30 Punkte, *TMT‑B*: in Sekunden, *LPS 50+*: ≥ 30 nicht pathologisch, *FAS*: ≥ 30 nicht pathologisch, *GDS*: Max. 15 Punkte

**Table 8:** 

Test	Mittelwert (SD) des ersten Kollektivs	Mittelwert (SD) des zweiten Kollektivs
O‑DEM-Gesamtscore	6,87 (3,0)	8,11 (1,9)
O‑DEM Supermarkt	2,68 (1,4)	3,49 (0,8)
O‑DEM TMT‑A	2,45 (1,7)	2,95 (1,2)
O‑DEM Subtraktion	1,74 (0,6)	1,53 (0,8)

*SD* Standardabweichung, *erstes Kollektiv*: Patient*innen ohne subjektive HB, *zweites Kollektiv*: Personen mit objektiven HB

Der O‑DEM-Gesamtscore korrelierte signifikant mit den anderen kognitiven Tests und dem Alter (*p* ≤ 0,001), jedoch nicht mit Bildung und dem Hören (Tab. 9).

**Table 9:** 

	HI-MoCA	TMT‑B	LPS 50+	FAS	Alter	Bildung	Hören rechts	Hören links
O‑DEM	0,53*	−0,40*	0,23*	0,46*	−0,25*	−0,05	−0,08	0,11

*p ≤ 0,001; zweiseitig

Zur Bestimmung der Sensitivität und Spezifität wurden in der ROC-Analyse Personen ohne und mit kognitiven Einschränkungen verglichen. Die AUC der O‑DEM- und HI-MoCA-Gesamtscores waren vergleichbar hoch (Tab. 10). Die Tab. 11 zeigt die Umrechnung der Rohwerte in O‑DEM-Werte. Bei zwei der drei O‑DEM-Subtests (Subtraktion, Supermarkt) lag kein signifikanter Alterseffekt vor, wodurch auf eine Alterskorrektur verzichtet wurde. Auch konnte kein signifikanter Bildungseffekt gezeigt werden.

**Table 10:** 

Cut-off	O‑DEM	HI-MoCA
LPS 50 + t-Wert = 30	0,86	0,88
TMT‑B MW + 1,5 SD = 184 s	0,85	0,80

*MW* Mittelwert, *SD* Standardabweichung

**Table 11:** 

		Rohwert	O‑DEM-Wert
Supermarkt	Punkte	≥ 20	4
		17–19	3
		14–16	2
		10–13	1
		< 10	0
TMT‑A	Sekunden	0–55	4
		56–65	3
		66–75	2
		76–85	1
		> 85	0
Subtraktion	Punkte	4–5	2
		3	1
		< 3	0

## Diskussion

Der erste Teil der Studie untersuchte, inwiefern der O‑DEM Menschen mit und ohne kognitive Beeinträchtigungen unterscheidet. Der O‑DEM wurde dafür an Menschen ohne selbstberichtete HB getestet, um erste Richtwerte zu entwickeln.

Kognition war mit den O‑DEM-Subtests assoziiert. Geminderte kognitive Leistungen korrelierten mit niedrigen Punkten in der Supermarkt- und Subtraktionsaufgabe sowie einer längeren Bearbeitungszeit des TMT‑A. Bildung zeigte keine klinisch relevante Korrelation mit den Subtests.

Die Ergebnisse zeigen, dass der O‑DEM zwischen Menschen mit keinen, leichten und mittleren bis ausgeprägten kognitiven Beeinträchtigungen differenziert. Die Testleistungen der Gruppen unterschieden sich in den O‑DEM-Subtests. Die AUC weist auf eine gute bis sehr gute Sensitivität und Spezifität der O‑DEM-Subtests hin. Die Transformation der Rohwerte in einen Gesamtscore stellte sich wie folgt dar: Patient*innen mit den stärksten kognitiven Beeinträchtigungen erzielten den niedrigsten Gesamtwert, Patient*innen ohne Beeinträchtigungen die höchste Punktzahl. Für den Gesamtscore liegen Cut-off-Werte vor, mit denen das Testergebnis einfach und effizient klassifizierbar ist.

Der zweite Teil der Studie diente der O‑DEM-Validierung an einer Kohorte mit objektiven HB. Hierfür wurde der O‑DEM mit dem HI-MoCA im Hinblick auf seine Diskriminierungsfähigkeit von Menschen mit und ohne kognitive Beeinträchtigungen verglichen. Zudem wurde eine Umrechnung der Rohwerte in die O‑DEM-Werte zur Verfügung gestellt.

Der O‑DEM-Gesamtscore war signifikant mit Kognition und Alter korreliert, jedoch nicht mit Bildung und Hören. Niedrigere Werte in kognitiven Tests waren mit schlechteren Leistungen im O‑DEM assoziiert. Die Ergebnisse der ROC-Analyse des O‑DEM-Gesamtscores deuten auf eine mit dem HI-MoCA vergleichbare gute bis sehr gute Sensitivität hin.

Die Relevanz eines schnellen Screenings, das zwischen kognitiven und keinen kognitiven Beeinträchtigungen differenzieren kann, wird angesichts einer zunehmenden Prävalenz von Demenzerkrankungen und einhergehender Schwerhörigkeit deutlich [13]. Bewährte kognitive Screeningverfahren wie der MMST und DemTect berücksichtigen diese auditiven Einschränkungen nicht. Für Schwerhörige adaptierte kognitive Tests wie der HI-MoCA oder DemTect Eye+Ear sind oftmals aufgrund ihrer Länge und Komplexität nicht im Alltag praktikabel.

### Limitationen

Im ersten Teil der Studie fehlen objektive Hördaten, daher kann das Vorliegen einer HB nicht mit Sicherheit ausgeschlossen werden. Weitere Limitationen sind die unterschiedliche Erfassung von Bildung und die verschiedenen Altersspannen im ersten (17 bis 94 Jahre) und zweiten Teil (51 bis 92 Jahre) der Studie.

### Stärken des O-DEM

1. Der O‑DEM ist in etwa 6 bis 8 min durchführbar (TMT-A: 2–5 min, Supermarktaufgabe: 2 min, Subtraktionsaufgabe: 1 min) und ist somit deutlich schneller umsetzbar als der DemTect Eye+Ear, MoCA-HI oder MMST. Aufgrund ähnlich hoher Sensitivität des HI-MoCA und des O‑DEM unterstreicht die Zeitersparnis den Mehrwert des O‑DEM als eine qualitativ gleichwertige Alternative zum HI-MoCA.
2. Die O‑DEM-Instruktionen sind leicht verständlich, gut vermittelbar und können auch ergänzend schriftlich für Menschen mit HB präsentiert werden.
3. Aufgrund der Komplexität, Kürze und Anzahl der Aufgaben ist für den O‑DEM kein geschultes Fachpersonal erforderlich. Somit kann der O‑DEM aufgrund der einfachen Durchführung in Hausarzt- und HNO-Praxen zu einer orientierenden Untersuchung des kognitiven Status genutzt werden.
4. Im O‑DEM werden gezielte Domänen eingegrenzt, welche wichtige Funktionen des Alltags darstellen und sich bereits in nationaler und internationaler Diagnostik bewährt haben. Die vollständige Ausführung der Aufgaben ist gegeben, was die Reliabilität und Validität der Subtests erhöht, während im HI-MoCA die getesteten Domänen (z. B. TMT‑B, Würfel zeichnen, Tiere benennen) nur verkürzt untersucht und die vollständigen Aufgaben nicht durchgeführt werden. Zudem beinhaltet der O‑DEM im Vergleich zum HI-MoCA den TMT‑A anstelle des TMT‑B. Der TMT‑B, bei dem Zahlen und Buchstaben alternierend verbunden werden müssen, führt durch seine erhöhte Schwierigkeit der Instruktionen und Durchführung vor allem bei älteren Menschen zu einer hohen Abbruchrate.
5. Eine weitere Stärke ist die deutsche Normierung, die bei bisherigen Tests (z. B. HI-MoCA) fehlt.
6. Die Objektivität der Durchführung und Auswertung ist gegeben, und der O‑DEM lässt sich problemlos in andere Sprachen übersetzen.

## Fazit für die Praxis

Der O‑DEM ist ein einfach und schnell durchführbares Demenzscreening, das durch die visuelle Stimuluspräsentation geeignet ist, auch bei Menschen mit HB mögliche kognitive Defizite zu erheben. Damit berücksichtigt der O‑DEM eine Zielgruppe, die trotz eines erhöhten Demenzrisikos bislang wenig im Fokus kognitiver Screenings stand. Er ersetzt jedoch keine neuropsychologische Testbatterie. Die O‑DEM-Subtests beruhen auf gängigen nationalen und internationalen neuropsychologischen Testbatterien und untersuchen relevante kognitive Domänen visuell mit hoher Objektivität. Die vorliegende Studie liefert erste Richtwerte.
